# Fundal Variant Adenomyomatosis of the Gallbladder: Report of Three Cases and Review of the Literature

**DOI:** 10.4021/jocmr2010.05.338w

**Published:** 2010-06-15

**Authors:** Abdullah Ozgonul, Muharrem Bitiren, Muhammet E. Guldur, Ozgur Sogut, Leman E. Yilmaz

**Affiliations:** a Department of General Surgery, Harran University, School of Medicine, Sanliurfa, Turkey; b Department of Pathology, Harran University, School of Medicine, Sanliurfa, Turkey; c Department of Emergency Medicine, Harran University, School of Medicine, Sanliurfa, Turkey; d Department of Pathology, Sanliurfa Research and Training Hospital, Sanliurfa, Turkey

## Abstract

Adenomyomatosis of the gallbladder (adenomyomas or adenomyomatous hyperplasia) is relatively uncommon tumor or tumor-like lesions that are found in 2% - 5% of all cholecystectomies. They may involve in the fundal localization as a second frequency and have a predilection for the middle and elderly-aged women. Adenomyomas are histologically characterized by epithelial proliferation and muscular hyperplasia of the gallbladder. The chronic irritation is mostly aetiological factor and rarely has malignant potential. In this paper, the authors present the clinicopathological features of three cases with adenomyomatosis of the gallbladder and discuss under the light of current literature because of the rarity of these conditions.

**Keywords:**

Gallbladder; Adenomyomatosis; Fundal variant

## Introduction

Adenomyomatosis of the gallbladder (adenomyoma or adenomyomatous hyperplasia) is a reactive, hamarthomatous malformation or non-neoplastic tumor-like lesion of gallbladder [1-3]. It is most commonly seen in the gallbladder [4], more frequently in the extrahepatic biliary tracts [2, 5, 6] and less commonly in the stomach [7] or in the small intestines [8]. Radiological and histopathological features were defined in 1960 by Jutras [9] using different terms such as hyperplastic cholecystosis, and in the following years, terms of ‘adenomyoma’ [5, 6, 10, 11], ‘adenomyomatosis’ [4, 12-21], and ‘adenomyomatous hyperplasia’ began to be used [22].

Adenomyomatosis has three morphological types according to the localization in the gallbladder wall: (a) segmental type is termed when the lesion is annular and separating the two compartments of the gallbladder; (b) fundal type is defined if the lesion is localized on the base of the gallbladder through the lumen in a hemispheric shape; (c) diffuse type is termed if it causes thickness in the gallbladder wall. Histologically, it is characterized by hyperplasia of the muscular layer and proliferation of the mucosal glandular structures [2-4, 13].

Herein, we describe three cases with the macroscopic and microscopic characteristics in addition to the diagnostic and differential diagnostic criteria of fundal type gallbladders adenomyomatosis as it is a rarely encountered entity.

## Case Report

In this study, the clinical and histopathological characteristics of two patients presenting to our university hospital, and of one patient presenting to a training and research hospital with the diagnosis of gallbladder adenomyomatosis were evaluated. The two cases were female, and were 54 and 73 years old, respectively. The lesion was located in the fundus of gallbladder in all cases. The reasons for admission were dyspepsia, abdominal pain, nausea and right flank pain that had been present for seven months to two years. The common finding in the physical examination was right upper abdominal pain. The examination findings of the other systems, hematological and biochemical parameters were unremarkable. Stones of variable numbers were detected in the gallbladder by abdominal ultrasonography. In one patient, a 1 x 1 cm mass with regular margins was detected, which protruded into the lumen in the fundus of the gallbladder, in addition to cholelithiasis. Two patients complaining of acute abdominal pain and nausea were clinically and radiologically diagnosed as cholelithiasis in emergency department of our university hospital. The other case was diagnosed as cholelithiasis and adenoma of the gallbladder via abdominal examination and ultrasound findings in the research and training hospital. Laparoscopic cholecystectomy was performed on all the patients.

**Figure 1.**
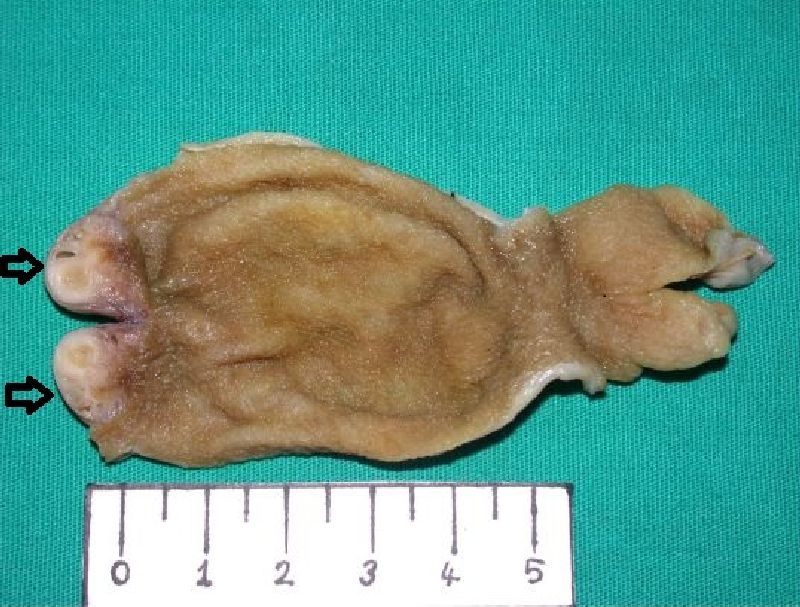
A white, firm hemisferic mass containing microcysts in the fundal region of the gallbladder (black arrows).

On macroscopic examination in all the cholecystectomy materials, gray-dull colored gallbladders were observed. When the gallbladders were dissected, a dark-green colored fluid and numerous yellow-brown gallstones of 0.3 - 1 cm diameter were demonstrated. The gallbladder lumens were smooth and the wall thickness was 0.2 - 0.3 cm. In two patients, a lesion of 0.7 cm diameter, and in one patient, a lesion of 1 cm diameter protruding into the lumen in the form of hemispherical bulging in the fundus of the gallbladder were detected. The sectional surfaces of these masses were gray-white colored and consisted of microcystic structures (Fig. 1).

**Figure 2.**
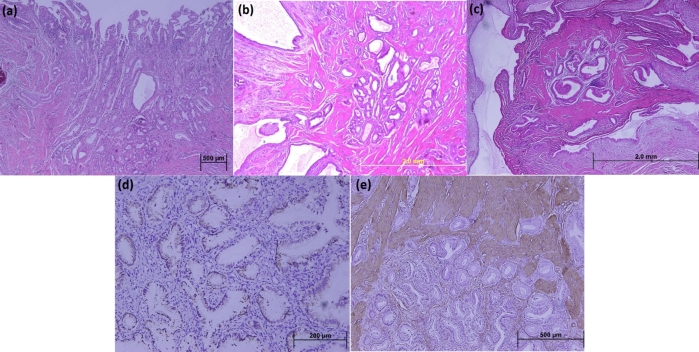
Photomicrographs of gallbladder sections: (a) Proliferation of the mucosal glandular structures inceptiving from the mucosa of gallbladder; (b) Hyperplasia of the smooth muscle cells among them (H&E stains; original magnifications, x40); (c) Aggregation of cystically dilated glandular structures surrounded by a hiperplasic smooth muscle tissu evaluate columnar without nuclear atypia in glandular epithelium. The glandular structures are lined by an unistratified epithelium at high magnification (H&E stain; original magnification, x100); (d) Immunohistochemical stains show the glandular columnar cells to be positive for cytokeratin-7 expression (original magnification, x200); (e) Immunohistochemical stains show the muscularis layer to be positive for alpha-smooth muscle actin expression ( original magnification, x100).

On microscopic examination, within a background of connective tissue, bundle-arranged smooth muscle cells, and numerous glandular lobules floored with columnar epithelial cells with nuclei located at the base were examined. Some of these glandular structures were cystically dilated and irregularly shaped, and no mitotic figures, atypical or pleomorphic changes were demonstrated (Fig. 2a, Fig. 2b, and Fig. 2c). Immunohistochemical stains performed on sections prepared from paraffin-embedded blocks using an avidin-biotin peroxidase complex method. The glandular epithelium revealed positive immunohistochemical reaction for cytokeratin-7 (CK; clone OV-TL12/30, dilution 1:50, DAKO), cytokeratin-20 (CK; clone Ks20.8, dilution 1:50, DAKO), (Fig. 2d). In addition, hyperplasic smooth muscle cell clusters stained diffusely positive for alpha-smooth muscle actin (Clone 1A4, dilution 1:40, DAKO) (Fig. 2e). These histopathological and immunohistochemical findings mentioned above were confirmed adenomyomatosis of the gallbladder in all cases.

## Discussion

In the series of Atsusi et al, an incidence of 34.5% was found for the fundal type of adenomyomatosis which was ranked second in the gallbladder, with the segmental type as 63.5%, and 2% for the diffuse type. The mean age of patients was 55.4 years and the range was 22 to 84 years [4]. The female/male ratio shows variation in different studies [3, 4, 13]. A few cases have been reported in the pediatric age group (range 15 to 17 years). All of our cases were female and the mean age was consistent with that in the literature. The histogenesis of adenomyomatosis is controversial [2]. First, the high rates of co-existence of adenomyomatosis with irritative conditions of the gallbladder such as chronic inflammation or cholelithiasis was remarkable, and it was suggested that it was a hyperplasic inflammatory lesion [4, 13]. Despite of being accepted as a hamartomatous lesion or pseudotumor [2], it has been reported that it may cause a benign tumor [22] or may be a pre-malignant lesion with the potential of developing into a malignant tumor in a few studies [18-21]. As biliary stasis occurs in the segmental type, the co-existence with cholelithiasis is the highest at 88.9%. This co-existence has been reported as 47.4% in the fundal and diffuse types [4]. All of our cases were fundal type adenomyomatosis, and they underwent laparoscopic cholecystectomy with the diagnosis of cholelithiasis.

There are no symptoms in adenomyomatosis of gallbladder except for a vague abdominal pain. The co-existence with cholelithiasis is another reason for its clinically silent characteristic and incidental detection after cholecystectomy [2, 4, 13]. Our cases were operated for cholelithiasis, and abdominal ultrasonography revealed a fundal mass lesion in one of our cases.

Most cases with adenomyomatosis of the gallbladder are diagnosed preoperatively using radiological investigations. Ultrasonography is the method of choice as it is inexpensive and practical. Dilated intramural cystic glands which are seen as artifacts and echogenic focuses causing focal or complete thickening in the gallbladder on ultrasonography are important findings for the diagnosis and are accepted as satisfactory for the preoperative differential diagnosis [4, 13, 21].

Fundal type adenomyomatosis is seen macroscopically as an intraluminal hemispheric mass in the fundus of the gallbladder. Section surfaces are hard and consist of gray-white tissue and between these, dilated cystic glands. Histological diagnosis is easy; it has distinguishing microscopic characteristics from other lesions. The cases with adenomyomatosis of the gallbladder have combinations of proliferated glandular lobules, cystically dilated structures, and smooth muscle cell bundles in the stroma which was composed of connective tissue. The proliferated glands are floored with cuboidal or cylindrical epithelial cells between dense interlacing smooth muscle cell bundles [1, 4, 13, 15]. These epithelial cells are positive for cytokeratin-7 and cytokeratin-20, similar to immunohistochemically normal biliary cells. The smooth muscle cell component is positive for alpha-smooth muscle actin [2, 5]. Our cases were assessed as fundal type adenomyomatosis with their macroscopic, microscopic and immunohistochemical characteristics. The differential diagnosis of fundal type adenomyomatosis consists of several lesions which cause wall thickness and which protrude into the lumen. The most dreaded is adenocarcinoma of the gallbladder. They are differentiated from adenomyomatosis with their infiltrative characteristics. Adenomas are benign tumors of the gallbladder requiring a differential diagnosis. Other common and protruding polypoid lesions of the gallbladder are adenomatous polyp, hyperplastic polyp, cholesterol polyps, and xanthogranulomatous cholecystitis. Neoplasms with mesenchymal origin such as neuroma, carcinoid tumor, leiomyoma, fibroadenoma, fibroma, and lipoma are other uncommon lesions requiring a differential diagnosis. Besides, metastatic diseases including malignant melanoma foci should be remembered in the differential diagnosis [1, 13, 20].

Fundal type adenomyomatosis is usually medically treated if the diagnosis has been made radiologically. If there is no response to medical treatment, cholecystectomy is the treatment of choice [2, 4, 14]. One of our patients was operated with the diagnosis of cholelithiasis and gallbladder adenoma. Incidental adenomyomatosis was found in the other two patients who were operated with the diagnosis of cholelithiasis.

In conclusion, the macroscopic and microscopic assessments of cholecystectomies which were performed for cholelithiasis in routine clinical studies are usually predictable. However, incidental fundal type adenomatosis may also be seen. Recognition of this rare entity is important because the similar characteristics with the adenoma and carcinoma of the gallbladder may confound the surgeons. These lesions should be kept in mind due to their rare potential of developing into benign or malignant tumor.
